# Study on serum miR-185-5p in assessing the injury severity and prognosis of patients with traumatic brain injury

**DOI:** 10.5937/jomb0-37716

**Published:** 2023-10-27

**Authors:** AiYu Chen, Xiang Tong, Tang LiZhen, Tao Lu, CaiHong Wu

**Affiliations:** 1 Chunan County Traditional Chinese Medicine Hospital, Department of Surgery, Hangzhou City, China; 2 Chun'an County Traditional Chinese Medicine Hospital, Department of Rehabilitation Medicine, Hangzhou City, China

**Keywords:** traumatic brain injury, miR-185-5p, injury severity, prognosis, biomarker, traumatska povreda mozga, miR-185-5p, težina povrede, prognoza, biomarker

## Abstract

**Background:**

This study aims to explore whether serum miR-185-5p levels are related to the injury severity and prognosis of traumatic brain injury patients.

**Methods:**

Serum miR-185-5p level was quantified in 120 TBI patients. The Glasgow Coma Scale (GCS) was used to grade the damage, and the Glasgow Outcome Scale (GOS) was used to evaluate the prognosis 3 months after TBI. Pearson correlation analysis was performed to determine the relationship between serum miR-185-5p level and injury severity and prognosis, and the value of serum miR-185-5p level to assess injury severity and prognosis was evaluated by receiver operating characteristic (ROC) curve.

**Results:**

Serum miR-185-5p level in moderate and severe TBI patients was higher than in mild TBI patients, and serum miR-185-5p was closely related to GCS score and GOS score. Serum miR-185-5p level higher than 0.36 could distinguish patients with mild to moderate TBI injury, with 72.97% sensitivity and 97.62% specificity, while that higher than 0.43 had 46.34% sensitivity and 91.89% specificity to distinguish moderate to severe TBI patients. Moreover, serum miR-185-5p levels higher than 0.36, with a sensitivity of 96.30% and a specificity of 60.24%, distinguished the poor prognosis of TBI patients. Serum miR185-5p level was an independent predictor of poor prognosis in TBI patients after 3 months and was effective in discriminating adverse outcomes at 3 months.

**Conclusions:**

Serum miR-185-5p level was significantly correlated with 3-month injury and adverse prognosis in TBI patients, suggesting that serum miR-185-5p level may be a biomarker that provides supplementary prognostic information and can be used to identify the risk of adverse prognosis in TBI patients.

## Abstract (sr)

**Uvod:**

Ova studija ima za cilj da istraži da li su nivoi miR185-5p u serumu povezani sa ozbiljnošću povrede i prognozom kod pacijenata sa traumatskim povredama mozga.

**Metode:**

Nivo miR-185-5p u serumu je kvantifikovan kod 120 pacijenata sa TBI. Za procenu oštećenja je korščena Glazgovska skala kome (GCS), a za procenu prognoze 3 meseca nakon TBI, Glazgovska skala ishoda (GOS). Urađena je Pirsonova korelaciona analiza da bi se odredio odnos između nivoa miR-185-5p u serumu i težine i prognoze povrede, a vrednost serumskog miR-185-5p nivoa za procenu ozbiljnosti povrede i prognoze je procenjena pomo u ROC krive.

**Rezultati:**

Nivo miR-185-5p u serumu kod pacijenata sa umerenom i te{kom TBI je bio viši nego kod pacijenata sa blagim TBI, a miR-185-5p u serumu je bio usko povezan sa GCS skorom i GOS skorom. Nivo miR-185-5p u serumu viši od 0,36 bi mogao da ukaže na distinkciju između pacijenata sa blagom do umerenom TBI povredom, sa 72,97% osetljivosti i 97,62% specifičnosti, dok je viši od 0,43 imao 46,34% osetljivosti i 91,89% specifičnosti u smislu distinkci je između pacijenata sa umerenom do te{kom TBI. Dodatno, nivoi miR-185-5p u serumu vi{i od 0,36, sa osetljivošću od 96,30% i specifičnošću od 60,24%, su ukazivali na lošu prognozu pacijenata sa TBI. Nivo miR-185-5p u serumu je bio nezavisni prediktor loše prognoze kod pacijenata sa TBI nakon 3 meseca i bio je efikasan u razlikovanju neželjenih ishoda nakon 3 meseca.

**Zaključak:**

Nivo miR-185-5p u serumu je u značajnoj korelaciji sa stanjem tri meseca nakon povrede i nepovoljnom prognozom kod pacijenata sa TBI, {to sugeriše da nivo miR-185-5p u serumu može da bude biomarker koji pruža dodatne prognostičke informacije i može da se koristi za identifikaciju rizika od loše prognoze kod pacijenata sa TBI.

## Introduction

Traumatic brain injury (TBI) is defined as »changes in brain function or other pathological changes caused by external forces« [1] [2] [3]. It is a neurosurgical disease with high fatality and disabilityrates, which can cause physical, cognitive, emotional, and behavioural symptoms [4] [5]. TBI is second only to cancer and cardiovascular disease in morbidity and mortality and is the leading cause of traumatic death [6] [7]. TBI, which affects an estimated 69 million people worldwide, is usually caused by traffic accidents, falling from high altitudes, extreme sports, and other violent hits to the head that cause short-term memory loss and motor and cognitive dysfunction [8] [9] [10]. If not diagnosed and treated in time, it can eventually cause irreversible neurological damage, seriously affecting the overall quality of life and reducing work ability [11]. At present, the clinical diagnosis of TBI is mainly based on the patient’s clinical symptoms, signs, neurological examinations (Glasgow Coma Scale, GCS), neuroimaging examinations (CT, MRI, SPECT), and laboratory examinations, including some brain tissue-specific proteins, such as glial fibrillary acidic protein, a microtubule-associated protein, neuron-specific enolase, S-100 calcium-binding protein, Tau protein, and ubiquitin C-terminal esterase L1 [12] [13] [14] [15] [16] [17]. However, due to the sensitivity of imaging techniques and the lack of specificity of diagnostic indicators, its differential diagnosis is still limited. As a result, there is a significant unmet medical need to identify accurate and widely available biomarkers to guide the diagnosis, prognosis, and treatment of patients with TBI [18].

At present, microRNAs (miRNAs) with tissue specificity, rapid release rate, and serum stability have been identified as non-invasive biomarkers in diagnosing and treating various diseases [19]. Although these molecules cannot encode proteins, they can regulate gene expression by degrading mRNA and inhibiting translation. miRNAs also play a crucial role in various physiological and pathological processes such as cell growth, differentiation, metabolism, apoptosis, angiogenesis, and tumorigenesis. miRNAs play an essential role in maintaining and regulating physiological functions in various diseases, including TBI, and therefore are receiving increasing attention in disease diagnosis and treatment targets [20] [21] [22]. miR-425-5p, miR-16, miR-92a, and miR-765 are abnormally expressed in patients with TBI and have high diagnostic values [23]. A recent study has also found 4 miRNAs (miR-203b-5p, miR-203a-3p, miR-206, and miR-185-5p) that can be used to identify TBI patients from healthy controls [24]. However, there is still no clear biomarker to diagnose TBI. The purpose of this study was to investigate the relationship between serum miR-185-5p level and TBI injury severity and prognosis, to screen out promising candidate biomarkers and provide useful information and direction for diagnosing TBI patients’ injury severity and prognosis.

## Materials and methods

### Research population

A total of 120 TBI patients treated in the Chunan County Traditional Chinese Medicine Hospital from June 2018 to June 2021 were included in the TBI group. Inclusion criteria: meeting TBI clinical diagnostic criteria; ≥ 18 years old; the window period from onset to admission is no more than 6 hours; Standardized treatment following international TBI guidelines [25]. Exclusion criteria: combined with brain tumours; severe heart, lung, liver, and kidney parenchymal diseases; blood system diseases; coagulation dysfunction [25]. This study was approved by the Chunan County Traditional Chinese Medicine Hospital ethics committee, and all subjects gave informed consent.

### Clinical data

Clinical data of all TBI patients were collected, including gender, age, comorbidities (hypertension and diabetes), unhealthy lifestyle habits (smoking and drinking), and causes of trauma (traffic accidents, accidental falls, and physical assault). The time from trauma to admission was recorded, and vital signs were measured, including systolic and diastolic blood pressure, heart rate, temperature, respiratory rate, and oxygen saturation. Radiological severity was evaluated by Rotterdam CT classification, including skulcap fracture, skull base fracture, epidural hematoma, subdural hematoma, ventricular haemorrhage, intracerebral hematoma, cerebral contusion, and subarachnoid haemorrhage. Venous blood was collected for laboratory tests, including white blood cells, blood glucose, serum C-reactive protein, D-dimer, fibrinogen, haemoglobin, platelets, sodium, potassium, prothrombin time, thrombin time, and partial thromboplastin time. Another blood sample was taken to detect serum miR-185-5p.

### Rating of damage

On admission, patients with TBI were given a Glas gow Coma Scale (GCS) score, and nervous system function was assessed by examining individual movements, speech, and eye-opening responses. 13–15 points indicated mild TBI, 9–12 points indicated moderate TBI, and 3–8 points indicated severe TBI [26].

### Evaluation of prognosis

All TBI patients were re-examined 3 months after injury, and the prognosis was evaluated by Glasgow Outcome Scale (GOS) score: satisfactory (5 points; recovery of learning ability and workability, potentially accompanied by mild dysfunction); moderate disability (4 points; self-care, but lack of behaviour, cognition and personality), severe disability (3 points; conscious, but severely impaired in language, cognitive, and physical functions), vegetative state (2 points; slow response, long-term coma, dementia or dementia cortical rigidity), death (1 point; stop of heartbeat and breathing). According to the GOS score, patients were divided into a good prognosis group (4–5 points), a poor prognosis group (2–3 points) and a death group (1 point) [27]. Fasting venous blood was collected from all subjects in the morning to detect serum miR-185-5p.

### Serum miRNA expression level

The collected blood was centrifuged at 3000 r/min for 10 minutes to separate the serum. Total serum RNA was extracted using miRNeasy Kit (Qiagen, GmbH, Hilden, Germany) and reversely transcribed using miRcute Plus miRNA First-Strand cDNA Synthesis Kit (Tiangen Biotechnology, Beijing, China) in the Proflex Base PCR system (Thermo Fisher Scientific, MA), USA). Total RNA was quantified using a Nanodrop 2000 spectrophotometer (Thermo Scientific, USA). miRNA was reverse transcribed into cDNA using Taq miRNA reverse transcription kit (Life Technologies; Thermo FisherScientific, Inc.). cDNA is used for each specific miRNA TaqMan assay. miRNA was quantified in the AB-7300 RT-PCR system (Thermo Fisher Scientific). miR-185-5p expression was standardized by U6.

### Statistical analysis

All statistical analyses were performed using SPSS 22.0 software. The measurement data were expressed as mean ± standard deviation, and the count data were expressed as the number of cases. Pearson correlation coefficient and receiver operating characteristic (ROC) curve were used to evaluate the correlation and predictive value of serum miR-185-5p with a 3-month prognosis. The maximum Youden index was calculated to determine the optimal cut-off value for prognosis, and its sensitivity and specificity were analyzed. *P* value <0.05 is considered significant.

## Results

### Comparison of clinical data

Among the 120 TBI patients, 64 were males, and 56 were females, ranging in age from 20 to 57 years old, with an average of (36.09±9.51) years. Among them, 35 cases had hypertension, 31 cases had diabetes, 30 cases had a smoking habit, 46 cases had a drinking habit; there were 63 cases of traffic accidents, 23 cases of accidental falls, 15 cases of beating injuries, and 19 other cases. According to the GCS score, TBI patients were divided into a mild group (42 cases), a moderate group (37 cases) and a severe group (41 cases). There was no statistical difference between the three groups in gender, age, comorbidities, unhealthy living habits, and causes of trauma (*p*>0.05), indicating comparability (Table 1, Table 2, and Table 3).

**Table 1:** Comparison of general information on TBI patients.

	Mild TBI (n=42)	Moderate TBI (n=37)	Severe TBI (n=41)	P value
Gender (male/female)	22/20	17/20	25/16	NS
Age (y)	37.14±9.49	36.08±9.83	35.02±9.36	NS
Complications				NS
Hypertension	17	11	17	
Diabetes mellitus	13	13	15	
Adverse life habits				NS
Cigarette smoking	10	8	12	
Alcohol consumption	13	17	16	
Traumatic mechanisms				NS
Traffic accident	18	21	24	
Accidental fall	7	8	8	
Assault	9	3	3	
Others	8	5	6	
Time from trauma to admission (h)	2.96±1.07	3.54±1.09	4.04±1.20	0.0002
Systolic arterial pressure (mmHg)	126.98±8.65	128.05±10.24	127.20±10.02	NS
Diastolic arterial pressure (mmHg)	68.33±6.72	66.97±6.29	68.17±6.00	NS
Heart rate (beats/minute)	60.19±7.35	59.16±8.31	62.17±9.21	NS
Respiratory rate (breaths/minute)	15.90±6.23	16.38±1.11	16.27±1.28	NS
Body temperature (°C)	36.25±0.36	36.32±0.52	36.36±0.46	NS
Blood oxygen saturation (%)	86.76±2.70	85.97±3.69	86.59±2.65	NS

**Table 2:** Comparison of CT parameters in TBI patients.

	Mild TBI<br>(n=42)	Moderate<br>TBI<br>(n=37)	Severe<br>TBI<br>(n=41)	P<br>value
Skull-cap fracture	32	30	35	NS
Skull-base<br>fracture	25	22	23	NS
Epidural<br>hematoma	23	22	22	NS
Subdural<br>hematoma	30	26	25	NS
Intraventricular<br>haemorrhage	5	7	4	NS
Intracerebral<br>hematoma	33	27	30	NS
Brain contusion	30	24	25	NS
Pneumocephalus	15	25	22	0.0175
Abnormal cisterns	21	33	37	<0.0001
Midline shift<br>> 5 mm	25	32	30	0.0275
Traumatic SAH	28	31	30	NS

**Table 3:** Comparison of laboratory indicators of TBI patients.

	Mild TBI (n=42)	Moderate TBI (n=37)	Severe TBI (n=41)	P value
Blood white blood cell (×10^9^/L)	6.85±0.77	7.77±0.66	8.34±0.61	<0.0001
Blood glucose (mmol/L)	10.04±2.01	11.17±1.93	14.17±1.90	<0.0001
Serum C-reactive protein (mg/L)	14.46±2.34	16.66±1.68	18.01±1.33	<0.0001
Serum D-dimer (mg/L)	4.20±0.60	4.33±0.63	4.48±0.51	NS
Serum fibrinogen (g/L)	3.09±0.58	3.23±0.49	3.00±0.45	NS
Blood hemoglobin (g/L)	118.38±9.00	122.05±8.40	127.20±9.43	<0.0001
Blood platelet (×10^9^/L)	158.19±9.73	162.70±11.76	165.17±14.18	0.0304
Blood sodium (mmol/L)	137.05±3.12	138.38±2.67	140.07±3.64	0.0002
Blood potassium (mmol/L)	3.97±0.32	4.09±0.37	4.04±0.35	NS
Prothrombin time (s)	13.89±0.82	14.55±0.88	14.47±0.95	0.0015
Thrombin time (s)	16.93±1.13	17.22±1.12	17.30±1.01	NS
Partial thromboplastin time (s)	38.19±2.68	39.19±2.81	39.25±2.88	NS

**Figure 1:**
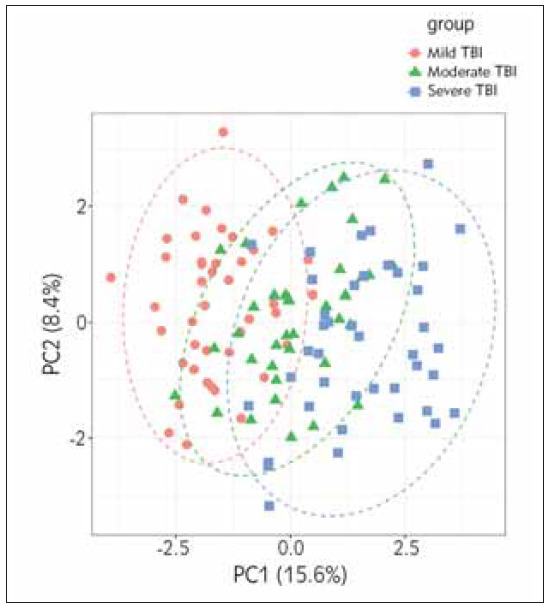
PCA analysis of three groups of patients based on clinical data.

PCA analysis found significant differences in clinical indicators, systolic blood pressure, diastolic blood pressure, heart rate, respiratory rate, body temperature, oxygen saturation, and laboratory indicators from trauma to admission (Figure 1). There were significant differences in the time from trauma to admission, white blood cells, blood glucose, serum C-reactive protein, haemoglobin, platelets, sodium, and prothrombin (Table 1, Table 2, and Table 3).

### The relationship between serum miR-185-5p level and clinical characteristics and injury severity in TBI patients

**Figure 2:**
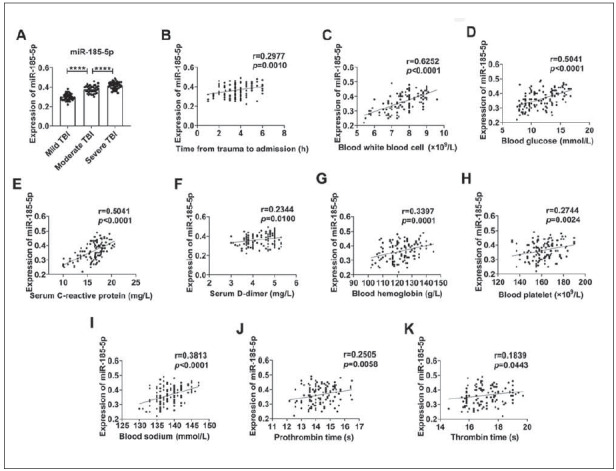
The relationship between serum miR-185-5p levels and clinical characteristics of TBI patients.

Serum miR-185-5p levels in moderate and severe groups were higher than those in the mild group, and serum miR-185-5p levels in the severe group were higher than those in the moderate group. The results indicated that serum miR-185-5p level was also increased with the aggravation of TBI (Figure 2A). Pearson correlation analysis was further conducted to study the relationship between serum miR-185-5p level and clinical characteristics of TBI patients. Serum miR-185-5p levels were correlated with time from trauma to admission, white blood cells, blood glucose, serum C-reactive protein, D-dimer, haemoglobin, platelets, sodium, prothrombin time, and thrombin time (Figure 2B-K).

**Figure 3:**
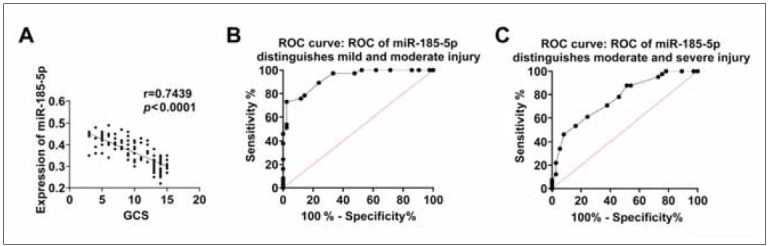
The relationship between the level of serum miR-185-5p and the injury severity in TBI patients.

In addition, serum miR-185-5p levels were negatively correlated with GCS scores. With the increaseof the GCS score, the serum miR-185-5p level decreased gradually (Figure 3A). ROC curve analysis confirmed the ability of serum miR-185-5p to distinguish the injury severity in TBI patients. Serum miR-185-5p levels higher than 0.36 could distinguish mild to moderate TBI patients, with sensitivity and specificity of 72.97% and 97.62%, respectively, and AUC of 0.9315 (95% confidence interval, 0.8802-0.9827; p<0.0001) (Figure 3B). Serum miR-185-5p levels higher than 0.43 could distinguish TBI patients with moderate and severe injury, with sensitivity and specificity values of 46.34% and 91.89%, respectively, and an AUC of 0.7680 (95 % Confidence interval, 0.6647–0.8712; p<0.0001) (Figure 3C).

Comparison of serum miR-185-5p levels in TBI patients ranked according to the injury severity. (B) The correlation between serum miR-185-5p level and the time from trauma to hospital admission. (C) The correlation between serum miR-185-5p and white blood cell levels. (D) Correlation between serum miR-185-5p and blood glucose level. (E) The correlation between serum miR-185-5p and serum C-reactive protein level. (F) Correlation between serum miR-185-5p and D-dimer levels. (G) Correlation between serum miR-185-5p and haemoglobin level. (H) Correlation between serum miR-185-5p and platelet level. (I) The correlation between serum miR-185-5p and serum sodium level. (J) Correlation between serum miR-185-5p level and prothrombin time. (K) Correlation between serum miR-185-5p level and thrombin time. ^****^ *p*<0.0001.

The correlation between serum miR-185-5p level and Glasgow Coma Scale score in TBI patients. (B) ROC curve of serum miR-185-5p level distinguishes mild and moderate injury in TBI patients. (C) The ROC curve of serum miR-185-5p levels distinguishes moderate and severe injury in TBI patients.

### The value of serum miR-185-5p level in predicting the prognosis of TBI patients

**Figure 4:**
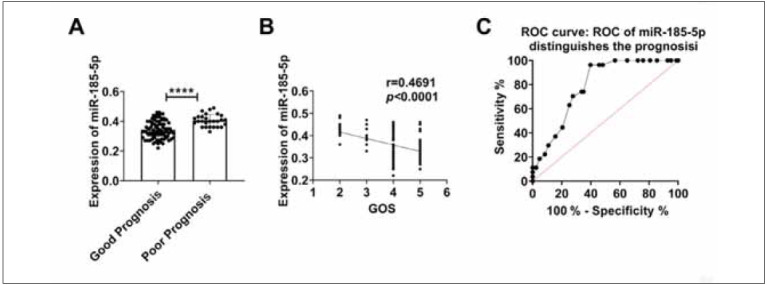
The relationship between serum miR-185-5p levels and the prognosis of TBI patients.

GOS score divided the patients into a good prognosis group (83 cases), a poor prognosis group (27 cases) and a death group (10 cases). Compared with the good prognosis group, serum miR-185-5p level in the poor prognosis group was increased (Figure 4A). In addition, serum miR-185-5p levels were negatively correlated with GOS scores. With the increase in GOS score, serum miR-185-5p level decreased gradually (Figure 4B). ROC curve analysis showed that serum miR-185-5p levels could significantly distinguish patients at a 3-month risk of poor prognosis. Serum miR-185-5p levels higher than 0.36 could identify patients with poor prognosis, with a sensitivity of 96.30%, specificity of 60.24%, and AUC of 0.7887 (95% confidence interval, 0.7055–0.8719; p<0.0001) (Figure 4C).

Comparison of serum miR-185-5p levels in TBI patients according to the prognostic effect rating. (B) Correlation between serum miR-185-5p level and prognostic effect score in TBI patients. (C) ROC curve of serum miR-185-5p level distinguishes the prognosis of TBI patients. ^****^ *p*<0.0001.

## Discussion

With the vigorous development of the social economy and the rapid development of the transportation and construction industry, traffic accidents, falling from high altitudes, and other accidents will inevitably increase year by year [28] [29] [30]. The incidence of systemic injury, especially craniocerebral injury, is rising yearly, leading to increasing rates of disability and fatality and bringing a massive burden to individuals, families, and society [31]. Accurate evaluation of traumatic brain injury is of great significance for diagnosis and treatment, and prognosis evaluation [32]. In this study, the predictive ability of serum miR-185-5p level on clinical damage and prognosis of TBI patients was evaluated, and it was found that serum miR-185-5p level was closely related to trauma severity of TBI patients. Elevated serum miR-185-5p levels were highly correlated with poor prognosis at 3 months. Serum miR-185-5p level may be a valuable biomarker for diagnosing damage and prognosis in TBI patients.

miRNAs exist stably in the systemic circulation of serum, plasma, saliva, urine, peritoneal fluid, pleural fluid, and milk [33] [34] [35]. miRNAs can bind to the 3’-UTR by complementary base pairing to inhibit protein transcription or promote mRNA degradation [36]. Studies have shown a correlation between miRNA and the occurrence and development of TBI [37] [38] [39]. It has been found that in the brain tissue of mice within 72 hours after TBI, miR-711 is up-regulated, protein kinase B is targeted to down-regulate, and the activities of FOXO3a/GSK3 and Caspase-3 are enhanced, suggesting that miR-711 could promote neuronal apoptosis after TBI [40]. miR-23a and miR-27a are down-regulated in the cerebral cortex of mice after TBI, and the expression of pro-apoptotic protein factors noxa, puma, Bax and BH3-noly is up-regulated, suggesting that miR-23a and miR-27a regulate cell apoptosis and nerve cell repair after TBI [41]. miR-185-5p (miR-185) was first identified as controlling the growth of human lung cancer cells [42]. miR-185-5p expression is up-regulated in fetuses with late-onset growth restriction, suggesting that miR-185-5p is a key regulator during brain development [43]. It has been confirmed that overexpression of miR-185-5p inhibits neuronal growth and promotes neuronal apoptosis in hypoxic-ischemic encephalopathy, leading to motor and cognitive deficits [44]. miR-185-5p is abnormally highly expressed in ischemic stroke and is correlated to infarct size and angiogenesis, serving as an independent diagnostic factor for acute ischemic stroke [45] [46]. In addition, Cheng et al. [47] demonstrated that increased miR-185-5p expression leads to the permeability of the blood-brain barrier. In addition, miR-185-5p could be a potential biomarker for clinical TBI to distinguish patients with TBI from healthy controls [24]. Therefore, miR-185-5p may be closely related to regulating neuronal proliferation, vascular regeneration, and blood-brain barrier function during TBI. In this study, our results showed that serum miR-185-5p levels were significantly elevated in moderate or severe TBI patients.

Subsequently, we analyzed the correlation between serum miR-185-5p levels and patient trauma severity. The results showed that serum miR-185-p increased with the severity of TBI injury, which is consistent with a previous study [47]. Lower blood sugar, haemoglobin, platelets, and sodium levels lead to lower brain oxygen levels, leading to a series of secondary nerve damage. Traumatic coagulopathy is associated with the severity of TBI. The C-reactive protein levels and white blood cells reflect the body’s inflammatory response and are closely related to the prognosis of TBI patients [48] [49] [50] [51] [52]. In this study, our results confirmed that serum miR-185-5p levels were significantly associated with time from trauma to admission, white blood cells, blood glucose, serum C-reactive protein, D-dimer, haemoglobin, platelets, blood sodium, and prothrombin time. The clinical indicators of TBI patients were closely related to the patient’s prognosis. Therefore, we next assessed the relationship between miR-185-5p and prognosis. The results showed that serum miR-185-5p levels were significantly higher in patients in the poor prognosis group compared to the good prognosis group. In conclusion, our study confirmed that miR-185-5p might be one of the promising biomarkers in TBI.

## Conclusion

In summary, our current study has determined that elevated serum miR-185-5p levels are highly correlated with injury severity and poor prognosis in TBI patients; namely, serum miR-185-5p can be used as a valuable biomarker to help assess TBI severity and predict prognosis. However, to further understand the complex relationship between TBI and candidate miRNAs, more attention needs to be paid to miRNA-related pathogenesis studies in TBI patients in the future.

## Dodatak

### Conflict of interest statement

All the authors declare that they have no conflict of interest in this work.
